# Red and processed meat consumption and gastric cancer risk: a systematic review and meta-analysis

**DOI:** 10.18632/oncotarget.15699

**Published:** 2017-02-25

**Authors:** Zhanwei Zhao, Zifang Yin, Qingchuan Zhao

**Affiliations:** ^1^ Xijing Hospital of Digestive Diseases, The Fourth Military Medical University, Xian, China; ^2^ Department of Obstetrics, Northwestern Women and Childrens Hospital, Shaanxi, China

**Keywords:** diet, gastric cancer, red meat, processed meat, meta-analysis

## Abstract

The associations between red and processed meat consumption and gastric cancer risk have remained inconclusive. We performed a systematic review and meta-analysis to analyze these associations. We searched PubMed and EMBASE to identify studies published from inception through October 2016. Subtype analyses of gastric cancer (gastric cardia adenocarcinoma and gastric non-cardiac adenocarcinoma) and dose-response analyses were performed. We finally selected 42 eligible studies. The summary relative risks of highest versus lowest consumption were positive for case-control studies with 1.67 (1.36-2.05) for red meat and 1.76 (1.51-2.05) for processed meat, but negative for cohort studies with 1.14 (0.97-1.34) for red meat and 1.23 (0.98-1.55) for processed meat. Subtype analyses of cohort studies suggested null results for gastric cardia adenocarcinoma (red meat, *P* = 0.79; processed meat, *P* = 0.89) and gastric non-cardiac adenocarcinoma (red meat, *P* = 0.12; processed meat, *P* = 0.12). In conclusion, the present analysis suggested null results between red and processed meat consumption and gastric cancer risk in cohort studies, although case-control studies yielded positive associations. Further well-designed prospective studies are needed to validate these findings.

## INTRODUCTION

According to the Global Cancer Statistics 2012, gastric cancer (GC) presents an enormous public health problem as the third most common cause of cancer death in males and the fifth in females, with approximately 1 million new cases and 723,100 deaths each year worldwide [1]. Considering the increasing trend in the incidence of GC and the high fatality, finding novel strategies to prevent this disease is an urgent need. An increasing number of studies have focused on dietary factors [2–5]. However, the associations between red and processed meat consumption and GC risk have remained inconclusive. Some studies have shown positive associations [6, 7] but others have provided null results [8, 9]. Additionally, there was insufficient evidence for subtype of GC (GCA: gastric cardia adenocarcinoma and GNCA: gastric non-cardiac adenocarcinoma). Thus, in consideration of the large burden of GC worldwide and the controversial evidence, we conducted a systematic review and meta-analysis with the following objectives: (1) to provide an update based on more sufficient evidence and a quantitative synthesis of the eligible data on the associations between red and processed meat consumption and the risk of GC; and (2) to provide more detailed evidence according to subtype analyses; and (3) to evaluate the dose-response association between red and processed meat consumption and GC risk.

## RESULTS

### Literature selection, study characteristics and quality scores

**Figure 1:**
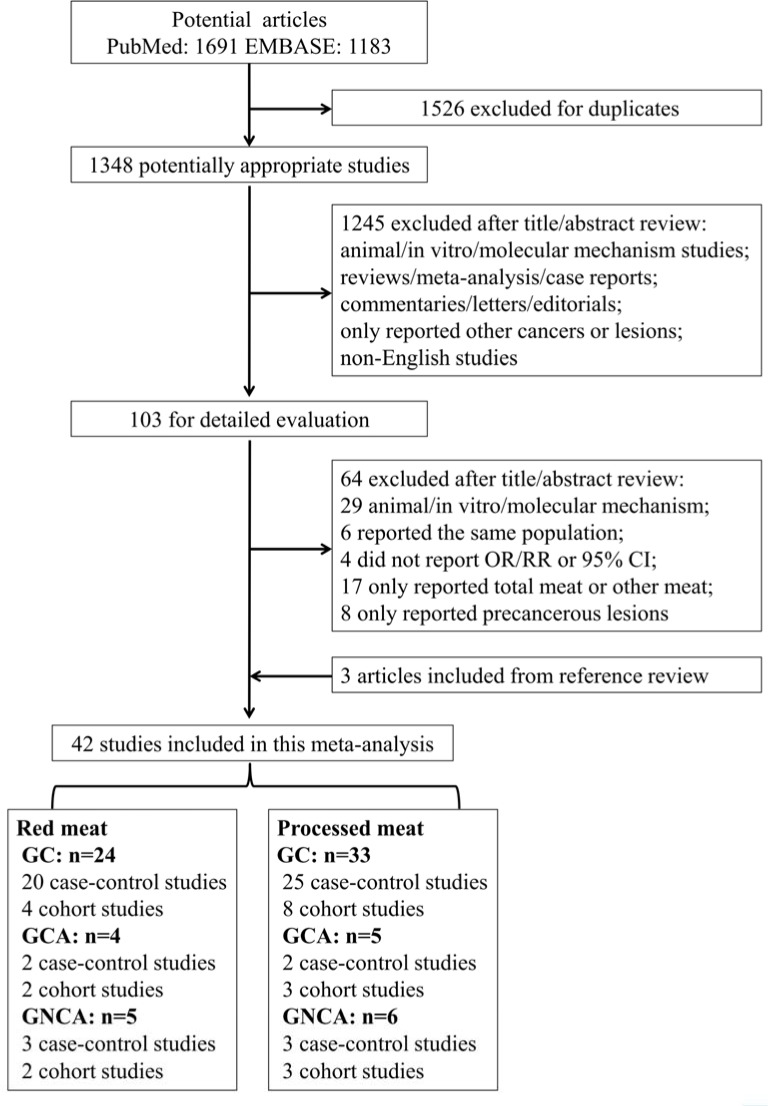
Flowchart of the process for the identification of relevant studies

Fourth-two studies met the eligibility criteria and provided 59 separate estimates (red meat = 24 and processed meat = 33) of the associations between red and processed meat consumption and GC risk (Figure 1). The selected studies were from 19 countries or regions in America, Europe and Asia with 805,890 participants and 9,851 cases for red meat consumption and 1,327,968 participants and 10,442 cases for processed meat consumption in relation to GC (Table 1).

**Table 1:** Baseline characteristics of included studies

First author, year, country	Study type	Case/control (cohort, n)	Study period	Method of dietary assessment	Type of dietary exposure	Dietary exposure categories	Adjusted RRs (95% CI)	Adjusted variables	NOS score
Risch 1985 Canada[1]	cc	246/246	1979-1982	FFQ-NS	Smoked/salted /picked meat	Tertile	3.92 (1.76-8.75)	Age, sex, ethnicity, intakes of grains, chocolate, fibrous foods, egg, and public water supply	6
La Vecchia 1987 Italy[2]	cc	206/474	1985-1986	FFQ-29	Ham	Tertile	1.60 (1.10-2.30)	Age, sex, education, residence and intakes of sugar, pasta/rice, polenta, whole grain bread/pasta, fruits and vegetables	6
Lee 1990 China[3]	cc	210/810	1954-1988	FFQ-NS	Cured meat	Tertile	2.31 (1.30-4.00)	age, sex, and hospital	5
Boeling1 1991 Germany[4]	cc	143/579	1985-1986	FFQ-74	Processed meat	Tertile	2.21 (1.32-3.71)	Age, sex, hospital, and intakes of cheese, whole meal bread, raw vegetables and citrus fruits	6
Boeling2 1991 Poland[5]	cc	741/741	1986-1990	FFQ-43	Sausage	Tertile	1.55 (1.07-2.26)	age, sex, education, occupation, and residence	7
Gonzalez 1991 Spain[6]	cc	354/354	1987-1989	FFQ-NS	Cured meat	Quartile	1.40 (0.80-2.20)	age, sex, and intakes of preserved fish, egg, nuts, fruits, vegetables, and energy	6
Hoshiyama 1992 Japan[7]	cc	294/294	1984-1990	FFQ-24	Smoked/bacon/ ham	Tertile	1.40 (0.90-2.40)	age, sex, residence, and smoking	5
Sanchez-Diez 1992 Spain[8]	cc	109/123	1975-1986 1987-1988	FFQ-NS	Smoked/ sausage	≥1 vs <1 daily	3.35 (1.59-7.94)	age, sex, and residence	5
Hansson 1993 Sweden[9]	cc	338/679	1989-1992	FFQ-45	Red meat Bacon	quartile	0.73 (0.45-1.20) 1.42 (0.90-2.23)	age, gender, SES	5
Nazario 1993 Puerto Rico[10]	cc	136/151	1984-1986	FFQ-NS	Bacon	High vs low	2.10 (1.20-3.50)	no	6
Munoz 1997 Italy[11]	cc	722/2024	1985-1992	FFQ-36	Red meat Canned meat	Tertile ≥1 vs <1 daily	3.38 (1.42-8.04) 1.90 (1.04-3.47)	sex, age, area of residence and education	6
Ji 1998 China[12]	cc	1124/1451	1988-1989	FFQ-74	Red meat	Quartile	0.90 (0.60-1.20)	age, income, education, smoking (males only) and alcohol drinking (males only)	7
Ward 1999 Mexico[13]	cc	220/752	1989-1990	HHHQ-NS	Beef/liver Processed meat	Quartile	3.10 (1.60-6.20) 3.20 (1.50-6.60)	age, gender, total calories, chili pepper, added salt, history of peptic ulcer, smoking, SES	7
Palli 2001 Italy[14]	cc	126/561	1985-1987	FFQ-181	Red meat Meat sauce	Tertile	4.10 (2.10-7.90) 4.20 (1.20-14.9)	age, sex, social class, family history of GC, residence, BMI, total energy, and consumption tertiles of each food	7
Chen 2002 USA[15]	cc	124/449	1988-1993	HHHQ-NS	Red meat Processed meat	Quintile	2.00 (0.85-4.70) 1.70 (0.72-3.90)	age, sex, energy intake, respondent type, BMI, alcohol, tobacco, education, family history, and vitamin	7
Kim 2002 Korea[16]	cc	136/136	1997-1998	FFQ-109	Beef	Tertile	1.67 (0.86-3.24)	sex, age, SES, family history and refrigerator use	6
Ito 2003 Japan[17]	cc	508/36490	1988-1998	FFQ-NS	Beef Processed meat	Quartile	0.97 (0.39-2.39) 0.98 (0.73-1.32)	age, year and season of first hospital-visit, smoking habit and family history	8
Lissowska 2004 Poland[18]	cc	274/463	1994-1996	FFQ-NS	Red meat Sausage	Quartile	1.51 (0.90-2.51) 1.23 (0.79-1.93)	age, sex, education, smoking, and calories from food	7
Fei 2006 China[19]	cc	189/567	1972-2001	FFQ-NS	Red meat	Quartile	2.61 (1.79-3.81)	age and sex	5
Phukan 2006 India[20]	cc	329/658	2001-2004	FFQ-NS	Beef Smoked/salted meat	Quartile	0.89 (0.03-9.40) 2.80 (1.70-8.80)	education, tobacco use, drinking, and each dietary variable for another	7
Strumylaite 2006 Lithuania[21]	cc	379/1137	2002-2004	ACCQ-56	Salted meat	Tertile	2.21 (1.43-3.42)	smoking, alcohol, family history, BMI, physical activity, diet (salt preserved food items, bread, noodles, rice, different dairy products, mayonnaise, eggs, carrots, cabbage, broccoli, tomatoes, garlic, onion, paprika, bean, potatoes)	7
Wu 2007 USA[22]	cc	623/1308	1992-1997	FFQ-124	Red meat Processed meat	Quartile	1.57 (1.00-2.40) 1.65 (1.10-2.50)	age, sex, race, birthplace, education, smoking, BMI, GR, use of vitamins, total calories, *H. pylori*	8
Hu 2008 Canada[23]	cc	1182/5039	1994-1997	FFQ-69	Red meat	Quartile	1.20 (1.00-1.50)	age, province, education, BMI, sex, alcohol, smoking, vegetable, fruit, and total energy intake	7
Aune 2009 Uruguay[24]	cc	275/2032	1996-2004	FFQ-64	Red meat	Tertile	2.19 (1.31-3.65)	age, sex, residence, education, income, interviewer, smoking, alcohol, dairy foods, grains, fatty foods, fruits and vegetables, fish, poultry, mate drinking, BMI and energy intake	7
Pourfarzi 2009 Iran[25]	cc	217/394	2004-2005	FFQ-NS	Red meat Processed meat	Tertile ≥1 vs <1 per month	3.40 (1.79-6.46) 1.14 (0.55-2.37)	gender, age, education, family history, citrus fruits, garlic, onion, fish, dairy products, strength and warmth of tea, preference for salt intake and *H. pylori*	7
Gao 2011 China[26]	cc	270/403	1997-2005	FFQ-NS	Red meat Salted meat	Tertile	1.77 (1.21-2.58) 1.46 (1.16-1.87)	age, gender, geographic region	6
Hu 2011 Canada[27]	cc	1182/5039	1994-1997	FFQ-69	Processed meat	Quartile	1.70 (1.30-2.20)	age, province, education, BMI, sex, alcohol, smoking, total vegetable and fruit, and total energy intake; adjusted for strenuous and moderate activity for colon and rectum cancer	8
De Stefani 2012 Uruguay[28]	cc	274/2532	1996-2004	FFQ-64	Processed meat	Tertile	4.51 (2.34-8.70)	age, residence, BMI, smoking, alcohol drinking, mate′consumption, total energy, total vegetables and fruits, total white meat and red meat intakes	8
Ward 2012 USA[29]	cc	154/449	1992-1994	HHHQ-NS	Red meat processed meat	Quartile	2.16 (1.06-4.38) 0.97 (0.51-1.85)	year of birth, gender, cigarettes, education, vitamin C, fiber, carbohydrate, total calories intake	7
Di Maso 2013 Italy[30]	cc	230/1259	1991-2009	FFQ-NS	Red meat	Tertile	1.38 (0.92-2.07)	age, sex, education, BMI, smoking, alcohol, vegetable and fruit	8
Zamani 2013 Iran[31]	cc	190/647	2004-2011	FFQ-116	Red meat	Quartile	1.87 (1.01- 3.47)	age, sex, energy intake, ethnicity, hot tea consumption, tooth brushing, smoking, SES, and vegetable and fruit	7
Lin 2014 China[32]	cc	107/209	2009-2010	FFQ-NS	Salted meat	Tertile	5.95 (1.33-25.62)	age, gender, BMI, education, income, family history of cancer, smoking, alcohol	6
Somi 2015 Iran[33]	cc	212/404	2009-2011	FFQ-NS	Red meat	Yes vs no	1.05 (0.67-1.64)	age, sex, BMI, educational level, smoking	7
Nomura 1990 USA[34]	co	150/7990	1965-1968	FFQ-17	Ham/bacon/ sausage	Tertile	1.30 (0.90-2.00)	age	7
Zheng 1995 USA[35]	co	26/34691	1986-1992	FFQ-127	Processed meat	≥13 vs <4.4 times/ month	2.20 (0.80-6.00)	age, education, smoking	6
Galanis 1998 USA[36]	co	108/11907	1975-1980	FFQ-13	Processed meat	Tertile	1.00 (0.60-1.70)	age, education, place of birth, and gender. smoking and alcohol (only men)	7
Kanekt 1999 Finland[37]	co	68/9989	1966-1972	FFQ-NS	Cured meat	Quartile	0.49 (0.22-1.06)	sex, age, municipality, smoking and energy intake	6
Sauvaget 2005 Japan[38]	co	1270/38540	1980-1999	FFQ-22	Beef/pork	≥5 vs <2 times/week	1.06 (0.85- 1.34)	age, sex, residence, education, radiation exposure, smoking	7
Gonzalez 2006 Europe[39]	co	330/521457	1992-1998	FFQ-266	Red meat Processed meat	Quartile	1.50 (1.02-2.22) 1.62 (1.08-2.41)	sex, height, weight, education, tobacco, physical activity, alcohol, energy intake, vegetable, citrus/non-citrus fruit intake	8
Larrson 2006 Sweden[40]	co	156/61433	1987-1997	FFQ-65	Red meat Processed meat	Tertile	1.07 (0.69-1.66) 1.66 (1.13-2.45)	age, education, BMI, and intakes of total energy, alcohol, fruit and vegetables	7
Corss 2007 USA[41]	co	658/494036	1995-1996	FFQ-124	Processed meat	Quintile	1.00 (0.78-1.30)	age, sex, education, marital status, family history of cancer, race, BMI, smoking, frequency of vigorous physical activity, total energy intake, alcohol intake, and fruit and vegetable	9
Keszei 2012 Netherlands[42]	co	652/120852	1986-2002	FFQ-150	Red meat Processed meat	Quintile	1.15 (0.77-1.71) 1.19 (0.78-1.79)	age, smoking, energy intake, BMI, non-occupational physical activity, alcohol, vegetable and fruit, education	9

Abbreviations: GC: gastric cancer; cc: case-control; co: cohort; RRs: relative risks (highest vs lowest categories); 95% CI: 95% confidence intervals; FFQ: food frequency questionnaire; HHHQ: health habits and history questionnaire; ACCQ: Aichi cancer center questionnaire; BMI: body mass index; GR: gastroesophageal reflux; SES: socio-economic status.

### Red meat

#### High *vs* low consumption

**Figure 2:**
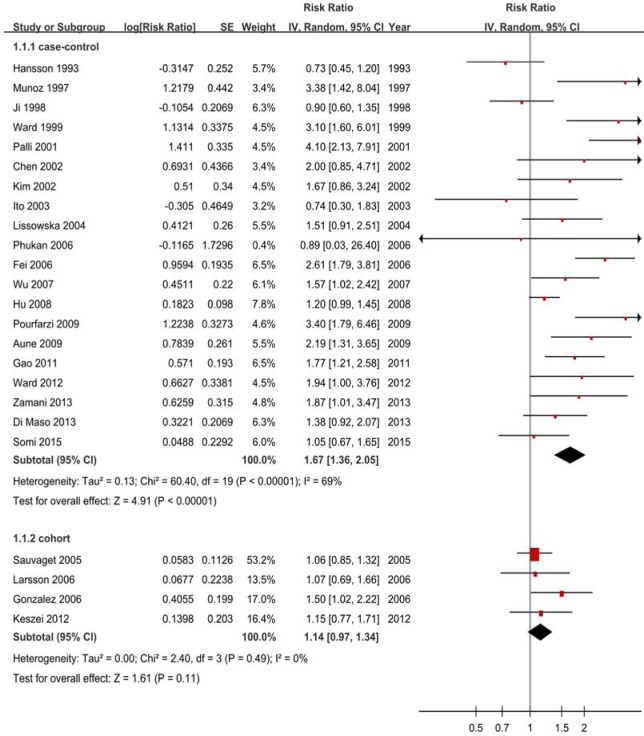
Forest plots of cohort studies for red meat consumption (highest ***vs*** lowest categories) and gastric cancer risk.

**Figure 3:**
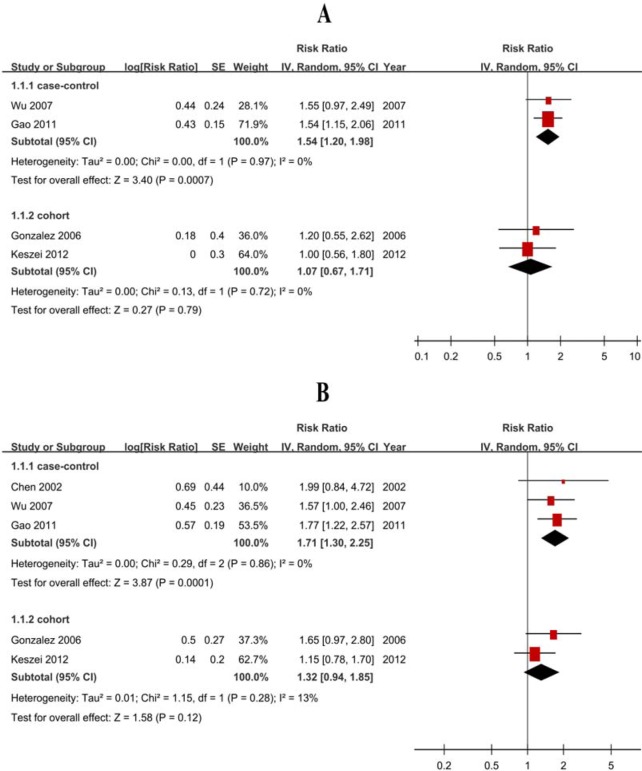
Forest plots of cohort studies for red meat consumption (highest *vs* lowest categories) and the risk of gastric cardia adenocarcinoma and gastric non-cardiac adenocarcinoma **A**. gastric cardia adenocarcinoma; **B**. gastric non-cardiac adenocarcinoma.

The pooled RRs were 1.67 (1.36-2.05) for case-control studies (Figure 2) but null results (RR = 1.14, 95% CI = 0.97-1.34) for cohort studies (Figure 2, Table 2). The subtype analyses showed negative for cohort studies, with 1.07 (0.67-1.71) GCA and 1.32 (0.94-1.85) for GNCA (Figure 3, Table 2).

**Table 2:** Subtype analyses of cohort studies for red and processed meat consumption (highest vs lowest categories) and the gastric cancer risk

Subtypes	Red meat					Processed meat				
	*n*	RR (95% CI)	*P*	*P*_h_	I^2^ (%)	*n*	RR (95% CI)	*P*	*P*_h_	*I*^2^ (%)
GC	4	1.14 (0.97-1.34)	.11	.49	0	8	1.23 (0.98-1.55)	.07	.09	43
GCA	2	1.07 (0.67-1.71)	.79	.72	0	3	1.03 (0.70-1.51)	.89	.22	35
GNCA	2	1.32 (0.94-1.85)	.12	.28	13	3	1.27 (0.94-1.70)	.12	.21	36

Abbreviations: GC: gastric cancer. GCA: gastric cardia adenocarcinoma. GNCA: gastric non-cardia adenocarcinoma. *P*: test for overall effect. *P*h: value for heterogeneity.

#### Heterogeneity

Although there was heterogeneity (*P* < 0.01, *I*^2^ = 69%) for case-control studies, there was no heterogeneity (*P* = 0.49, *I*^2^ = 0%) between cohort studies (Figure 2).

#### Publication bias

Tests of publication or small study bias were not conducted due to the small number of cohort studies. The sensitivity analysis of included cohort studies showed that the changes in recalculated RRs were not significant, with a range from 1.08 (0.90-1.29) when excluding Gonzalez 2006 (5.2%) to 1.24 (0.98-1.57) when excluding Sauvaget 2005 (6.4%).

#### Dose-response analysis

Four cohort studies were included, and the pooled RR was 1.12 (0.96-1.31) without heterogeneity (*P* = 0.64, *I*^2^ = 0%) for 100 g/day increase. The sensitivity analyses showed that the changes in the recalculated RRs were not significant, with a range from 1.07 (0.89-1.28) when excluding Larsson 2006 (25.7%) to 1.17 (0.98-1.40) when excluding Keszei 2012 (21.8%). The results demonstrated that a non-significant positive association was observed for EC risk. A non-linear dose-response analysis was not conducted due to the small number of included studies.

### Processed meat

#### High *vs* low consumption

**Figure 4:**
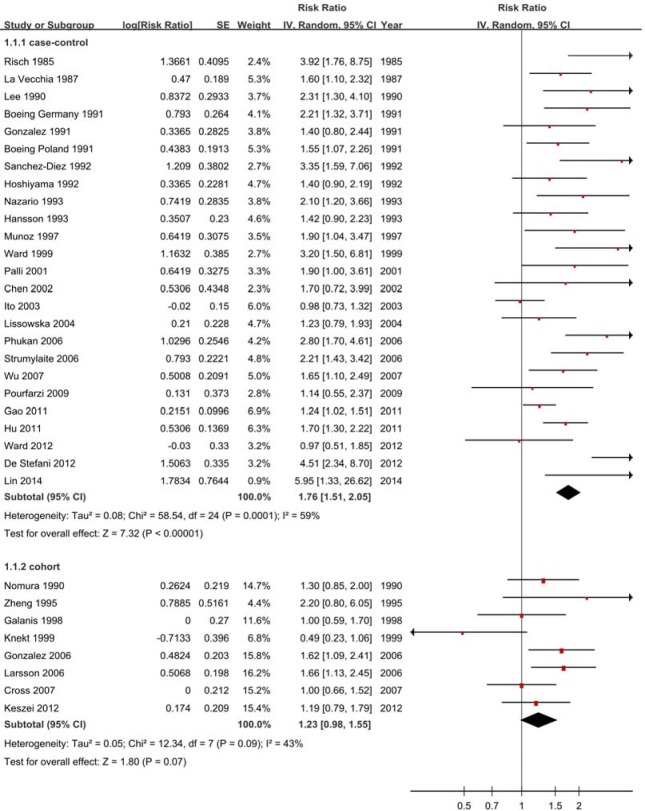
Forest plots of cohort studies for processed meat consumption (highest ***vs*** lowest categories) and gastric cancer risk.

**Figure 5:**
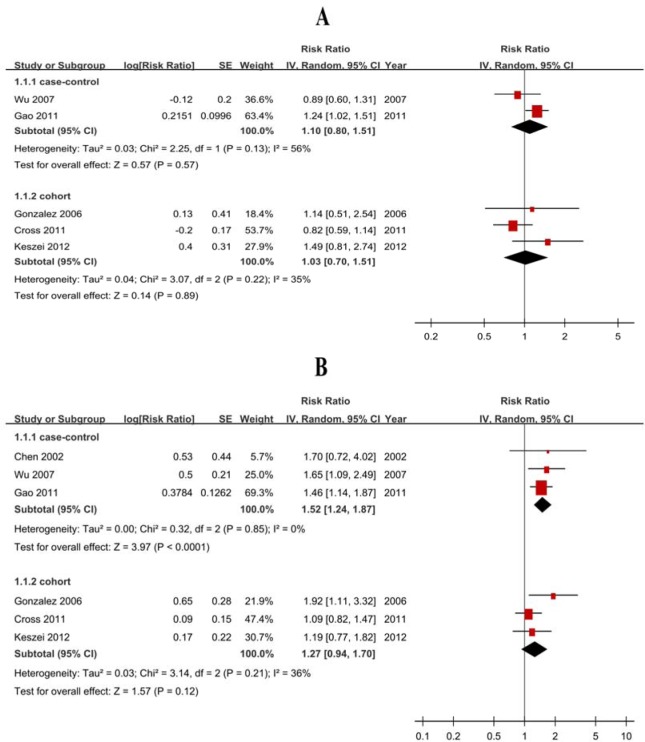
Forest plots of cohort studies for processed meat consumption (highest ***vs*** lowest categories) and the risk of gastric cardia adenocarcinoma and gastric non-cardiac adenocarcinoma. **A**. gastric cardia adenocarcinoma; **B**. gastric non-cardiac adenocarcinoma.

The pooled RRs were 1.76 (1.51-2.05) for case-control studies (Figure 4) but null results (RR = 1.23, 95% CI = 0.98-1.55) for cohort studies (Figure 4, Table 2). Subtype analyses showed negative for cohort studies, with 1.03 (0.70-1.51) GCA and 1.27 (0.94-1.70) for GNCA (Figure 5, Table 2).

#### Heterogeneity

There was heterogeneity (*P* < 0.01, *I*^2^ = 59%) for case-control studies and low heterogeneity (*P* = 0.09, *I*^2^ = 43%) between cohort studies (Figure 4).

#### Publication bias

**Figure 6:**
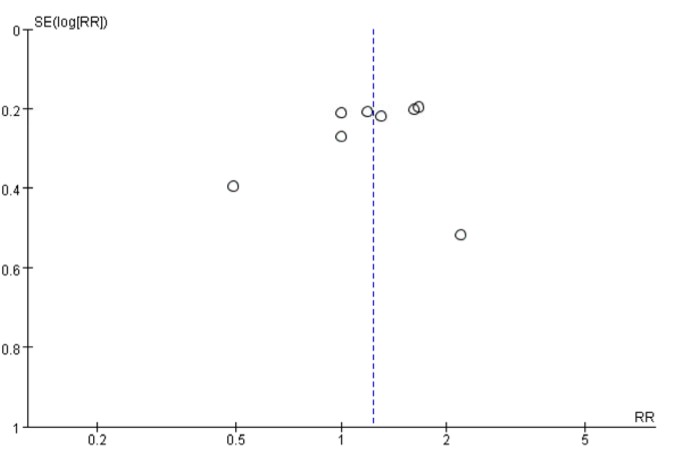
A funnel plot evaluating publication bias of studies for processed meat consumption and gastric cancer risk

A funnel plot, Begg’s test and Egger’s test were used to assess publication bias. The results of funnel plot (Figure 6), Egger’s test (*P* = 0.92) and Begg’s test (*P* = 0.71) indicated no evidence of publication bias. However, the sensitivity analysis of included cohort studies showed that the changes in the recalculated RRs were significant, with a range from 1.15 (0.91-1.44) when excluding Larsson 2006 (3.7%) to 1.26 (1.05-1.52) when excluding Knekt 1999 (1.9%).

#### Dose-response analysis

Seven cohort studies were included, and the pooled RR was 1.21 (1.04-1.41) without heterogeneity (*P* = 0.43, *I*^2^ = 0%) for 50 g/day increase. Nevertheless, the sensitivity analysis showed significant changes in the recalculated RRs, with a range from 1.28 (0.99-1.66) when excluding Gonzalez 2006 (58.4%) to 1.25 (1.06-1.47) when excluding Cross 2011 (14.0%). Additionally, non-linear associations were explored and the analysis did not suggest significant evidence of non-linear dose-response between processed meat consumption and GC (*P*_for nonlinearity_ = 0.13).

## DISCUSSION

Our findings provided detailed evidence that although high consumption of red and processed meat increases GC risk in case-control studies, positive associations were not observed in cohort studies. Similarly, the subtype analyses also showed that red or processed meat consumption was negatively associated with the risk of GCA and GNCA in cohort studies. The dose-response analyses found negative association for red meat and positive association for processed meat. Nevertheless, the sensitivity analysis of dose-response showed significant changes in the recalculated RRs. Overall, our detailed findings clarify the associations between red and processed meat consumption and GC risk, which provide valuable detail to update the dietary recommendations.

Several potential mechanisms may contribute to the effects. First, the positive results in the case-control studies may be biologically plausible. When cooked at high temperature for a long time, red and processed meat is a major source of carcinogens, including polycyclic aromatic hydrocarbons, heterocyclic amines and N-nitroso compounds, which may play important roles in the development of GC [13–15]. Second, a high iron intake associated with red and processed meat consumption may also play a role in GC by causing oxidative damage and involving the endogenous formation of carcinogenic *N*-nitroso compounds. Third, positive associations have been reported to be due to genetical differences [18]. Finally, bacteriological evidence has found possible mechanisms that explain the positive associations to a certain extent. Helicobacter pylori (*H. pylori)* has been deemed to be a significant risk factor of GC and has been classified as a type 1 carcinogen for humans by the International Agency for Research on Cancer (IARC) and the World Health Organization (WHO) [19]. Heme iron from red and processed meat may play an important role in GC risk by causing oxidative damage, which is considered to be an essential growth factor for *H. pylori* [16]. Nevertheless, the results of many cohort studies and meta-analyses do not support these explanations. For example, although consumption of red and processed was considered to be a risk factor for carcinogenesis, our previous findings did not support positive associations in some precancerous lesions [20] or cancer [21]. Furthermore, several large prospective investigations into cancer and nutrition suggested no potential association between higher consumption of red and processed meat and the risk of GC [8, 22]. Additionally, although studies showed positive associations between red meat consumption and gastrointestinal cancer, the definition of red meat included processed red meat, which may have contributed to the positive associations partly of red meat consumption [13, 23]. Thus, further studies are needed to verify these potential mechanisms.

## STUDY STRENGTHS AND LIMITATIONS

Our study has several strengths. The first strength is that we presented separate analyses according to study design and the subtypes of three cancers. These independent analyses provided detailed data and increased the power of the meta-analysis, which further strengthened the conclusion. Our analysis is based on a substantial sample size and a quantitative synthesis of the eligible data. These data provided sufficient reliable, robust and current evidence and increased the statistical power of the analysis. We broadly and systematically reviewed databases for all investigations of red and processed meat consumption and GC risk from database inception through October 2016, identifying all major published studies. The study selection and data extraction were performed independently and in duplicate by two investigators, which increased the validity of the results. Additionally, studies were identified from 19 countries or regions in the Americas, Europe and Asia, which increased the generalizability. Furthermore dose-response analyses were conducted to assess these associations rather than simply performing categorical comparisons.

However, the limitations of the present meta-analysis should be taken into consideration. First, the included studies were observational, and residual confounding and unmeasured factors cannot be excluded. Nevertheless, most included studies were adjusted for potential confounders, including sex, age, energy intake, body mass index, physical activity, smoking and alcohol use. Yet, information on some of the major confounders could still not be obtained from some of the studies. In particular, most of the included studies in relation to GC lacked information concerning *H pylori* infection. Only two studies adjusted the results modified by *H pylori* infection. Thus, the parts of the results should be considered with caution due to possible confounding.

Second, significant heterogeneity was observed in the included studies, which may be related to the publication year, number of cases, geographic region, method of exposure measurement, quality score and the different consumption levels of red and processed meat in studies. Nevertheless, heterogeneity was observed mainly in case-control studies and no statistically high heterogeneity was found in cohort studies. Due to many case-control studies providing exposure information obtained after the cancer diagnosis, which may be subject to inaccurate measurement of dietary intake and recall bias. Thus, the results of retrospective studies should not be overemphasized, and the results of prospective studies may be more powerful than retrospective studies. Additionally, we used random-effects models to account for heterogeneity.

Third, the results of the present study may have been influenced by publication bias. Indeed, Tests of publication or small study bias were not conducted due to the small number of cohort studies for several analyses and the corresponding results should be carefully interpreted.

Finally, we did not perform a subtype analysis of red and processed meat (e.g., beef, pork, mutton, bacon, ham and sausage). Our study did not investigate the associations of GC risk with other dietary factors, such as meat cooking techniques and heme iron from meat.

In conclusion, the present analysis suggested null results between red and processed meat consumption and GC risk in cohort studies, although case-control studies yielded positive associations. Further well-designed prospective studies are needed to validate these findings.

## MATERIALS AND METHODS

### Selection criteria

The selection criteria were as follows: histological features that were not consistent with the diagnostic gold standard were excluded; data that were incomplete or could not be combined were excluded; letters, comments, case reports, editorials, systematic reviews and meta-analyses, narrative reviews and studies in which only the abstract could be obtained were excluded; white meats, including poultry and fish, were excluded; total meats without citing red or processed meat were excluded; gastrointestinal stromal tumors, polyps, adenoma, precancerous lesions were excluded; the language of all studies was limited to English; and the studies were limited to those involving humans.

### Search strategy

We searched PubMed and EMBASE for studies published from inception through October 2016. The following search terms were used: “meat/meats”, “beef”, “veal”, “pork”, “lamb”, “mutton”, “ham”, “bacon”, “sausage”, “salami”, “hot dogs”, “diet/dietary” and “food/foods” in combination with *“gastrointestinal/digestive/alimentary tract/gastric/stomach”, “neoplasia/cancer/carcinoma/adenocarcinoma”*. The reference lists of the included studies were also searched manually to identify additional literature. The two sets of keywords were combined individually, and the eligibility criteria were independently judged by two authors (ZZ and ZY).

### Definitions and standardizations

#### Red and processed meat

In this study, red meat included beef, pork, lamb, mutton, beef burgers, veal, horse, liver and others. Processed meat included bacon, bacon rashers, lunch meat, ham, sausage, salami, hot dogs, souse meat, smoked meat, salted meat and others.

#### The subtypes of gastric cancer

Gastric cancer was subdivided into gastric cardia adenocarcinoma (GCA) and gastric non-cardiac adenocarcinoma (GNCA) based on the anatomic location.

#### Study quality

The Newcastle-Ottawa Scale (NOS) was used to assess the study quality of included studies [10]. The NOS is judged on three factors including the elucidation of the exposure or outcomes of interest for case-control or cohort studies, the selection of the study populations and the comparability of the populations. Two researchers (ZZ and ZY) independently assessed the quality of the studies, and discrepancies in interpretation were resolved by a consensus decision made by the third researcher (QZ). The range of NOS is 0-9 stars and a high quality study includes 7 or more stars.

### Data extraction

A data extraction sheet was generated for each included study and included the first author, publication year, country, study type, study population, study period, method of dietary assessment, dietary exposure categories, type of dietary exposure measured, adjusted RR (95% CI) (highest to lowest), adjusted variables and NOS score.

### Statistical analysis

The data were collected and extracted using SPSS 17.0 (Chicago, Illinois, USA). The RevMan5.3 (The Cochrane Collaboration, Oxford, UK) and STATA version 12.1 (STATA Corporation, College Station, TX) software were used for the data synthesis and analysis.

Random-effects models were used to pool the summary relative risks (RRs) and 95% confidence intervals (95% CIs). Heterogeneity among the studies was detected using the Q statistic (*P* < 0.1 was considered representative of significant heterogeneity) and the *I*^2^ statistics (*I*^2^ < 50% was considered low heterogeneity, and *I*^2^ > 50% was considered to indicate substantial heterogeneity) [11].

Publication bias was assessed using funnel plots, Begg’s test and Egger’s test (*P* < 0.1 was considered significant publication bias). The sensitivity analysis was conducted to investigate the influence of a specific study on the pooled risk estimate by removing one study in each round.

## References

[1] Torre LA, Bray F, Siegel RL, Ferlay J, Lortet-Tieulent J, Jemal A (2015). Global cancer statistics, 2012. CA Cancer J Clin.

[2] Fang X, Wei J, He X, An P, Wang H, Jiang L, Shao D, Liang H, Li Y, Wang F, Min J (2015). Landscape of dietary factors associated with risk of gastric cancer: A systematic review and dose-response meta-analysis of prospective cohort studies. Eur J Cancer.

[3] Hsiung HY, Fann JC, Yen AM, Chen SL, Chiu SY, Ku TH, Liu TY, Chen HH, Lin MW (2016). Stage-specific Dietary Factors Associated with the Correa Multistep and Multifactorial Process of Human Gastric Carcinogenesis. Nutr Cancer.

[4] Jeong M, Park JM, Han YM, Park KY, Lee DH, Yoo JH, Cho JY, Hahm KB (2015). Dietary prevention of Helicobacter pylori-associated gastric cancer with kimchi. Oncotarget.

[5] Lei L, Yang Y, He H, Chen E, Du L, Dong J, Yang J (2016). Flavan-3-ols consumption and cancer risk: A meta-analysis of epidemiologic studies. Oncotarget.

[6] González CA, Jakszyn P, Pera G, Agudo A, Bingham S, Palli D, Ferrari P, Boeing H, del Giudice G, Plebani M, Carneiro F, Nesi G, Berrino F (2006). Meat intake and risk of stomach and esophageal adenocarcinoma within the European Prospective Investigation Into Cancer and Nutrition (EPIC). J Natl Cancer Inst.

[7] Lin SH, Li YH, Leung K, Huang CY, Wang XR (2014). Salt processed food and gastric cancer in a Chinese population. Asian Pac J Cancer Prev.

[8] Keszei AP, Schouten LJ, Goldbohm RA, van den Brandt PA (2012). Red and processed meat consumption and the risk of esophageal and gastric cancer subtypes in The Netherlands Cohort Study. ANN ONCOL.

[9] Somi MH, Mousavi SM, Naghashi S, Faramarzi E, Jafarabadi MA, Ghojazade M, Majidi A, Naseri AS (2015). Is there any relationship between food habits in the last two decades and gastric cancer in North-Western Iran?. Asian Pac J Cancer Prev.

[10] Stang A (2010). Critical evaluation of the Newcastle-Ottawa scale for the assessment of the quality of nonrandomized studies in meta-analyses. EUR J EPIDEMIOL.

[11] Higgins JP, Thompson SG, Deeks JJ, Altman DG (2003). Measuring inconsistency in meta-analyses. BMJ.

[12] Egger M, Davey SG, Schneider M, Minder C (1997). Bias in meta-analysis detected by a simple, graphical test. BMJ.

[13] Cross AJ, Freedman ND, Ren J, Ward MH, Hollenbeck AR, Schatzkin A, Sinha R, Abnet CC (2011). Meat consumption and risk of esophageal and gastric cancer in a large prospective study. Am J Gastroenterol.

[14] Larsson SC, Bergkvist L, Wolk A (2006). Processed meat consumption, dietary nitrosamines and stomach cancer risk in a cohort of Swedish women. Int J Cancer.

[15] Samraj AN, Pearce OM, Laubli H, Crittenden AN, Bergfeld AK, Banda K, Gregg CJ, Bingman AE, Secrest P, Diaz SL, Varki NM, Varki A (2015). A red meat-derived glycan promotes inflammation and cancer progression. Proc Natl Acad Sci U S A.

[16] Ward MH, Cross AJ, Abnet CC, Sinha R, Markin RS, Weisenburger DD (2012). Heme iron from meat and risk of adenocarcinoma of the esophagus and stomach. Eur J Cancer Prev.

[17] Bastide NM, Chenni F, Audebert M, Santarelli RL, Tache S, Naud N, Baradat M, Jouanin I, Surya R, Hobbs DA, Kuhnle GG, Raymond-Letron I, Gueraud F (2015). A central role for heme iron in colon carcinogenesis associated with red meat intake. CANCER RES.

[18] Palli D, Russo A, Ottini L, Masala G, Saieva C, Amorosi A, Cama A, D’Amico C, Falchetti M, Palmirotta R, Decarli A, Mariani Costantini R, Fraumeni JF (2001). Red meat, family history, and increased risk of gastric cancer with microsatellite instability. Cancer Res.

[19] Uemura N, Okamoto S, Yamamoto S, Matsumura N, Yamaguchi S, Yamakido M, Taniyama K, Sasaki N, Schlemper RJ (2001). Helicobacter pylori infection and the development of gastric cancer. N Engl J Med.

[20] Zhao Z, Pu Z, Yin Z, Yu P, Hao Y, Wang Q, Guo M, Zhao Q (2016). Dietary fruit, vegetable, fat, and red and processed meat intakes and Barrett’s esophagus risk: a systematic review and meta-analysis. Sci Rep.

[21] Zhao Z, Yin Z, Pu Z, Zhao Q (2016). Association Between Consumption of Red and Processed Meat and Pancreatic Cancer Risk-a Systematic Review and Meta-analysis. Clin Gastroenterol Hepatol.

[22] Sauvaget C, Lagarde F, Nagano J, Soda M, Koyama K, Kodama K (2005). Lifestyle factors, radiation and gastric cancer in atomic-bomb survivors (Japan). Cancer Causes Control.

[23] Li WQ, Park Y, Wu JW, Ren JS, Goldstein AM, Taylor PR, Hollenbeck AR, Freedman ND, Abnet CC (2013). Index-based dietary patterns and risk of esophageal and gastric cancer in a large cohort study. Clin Gastroenterol Hepatol.

[24] Pourfarzi F, Whelan A, Kaldor J, Malekzadeh R (2009). The role of diet and other environmental factors in the causation of gastric cancer in Iran—a population based study. Int J Cancer.

[25] Wu AH, Tseng CC, Hankin J, Bernstein L (2007). Fiber intake and risk of adenocarcinomas of the esophagus and stomach. Cancer Causes Control.

